# Expansin-like Exl1 from *Pectobacterium* is a virulence factor required for host infection, and induces a defence plant response involving ROS, and jasmonate, ethylene and salicylic acid signalling pathways in *Arabidopsis thaliana*

**DOI:** 10.1038/s41598-020-64529-9

**Published:** 2020-05-08

**Authors:** Delia A. Narváez-Barragán, Omar E. Tovar-Herrera, Martha Torres, Mabel Rodríguez, Sonia Humphris, Ian K. Toth, Lorenzo Segovia, Mario Serrano, Claudia Martínez-Anaya

**Affiliations:** 1 Instituto de Biotecnología, Universidad Nacional Autónoma de México, 62210, Cuernavaca, Morelos Mexico; 2 Centro de Ciencias Genómicas. Universidad Nacional Autónoma de México, 62110 Cuernavaca, Morelos Mexico; 3 The James Hutton Institute, Invergowrie, Dundee DD2 5DA UK; 4 Present Address: Department of Biomolecular Sciences, The Weizmann Institute of Science, 7610001 Rehovot, Israel

**Keywords:** Biochemistry, Microbiology, Molecular biology, Plant sciences

## Abstract

Expansins are encoded by some phytopathogenic bacteria and evidence indicates that they act as virulence factors for host infection. Here we analysed the expression of *exl1* by *Pectobacterium brasiliense* and *Pectobacterium atrosepticum*. In both, *exl1* gene appears to be under quorum sensing control, and protein Exl1 can be observed in culture medium and during plant infection. Expression of *exl1* correlates with pathogen virulence, where symptoms are reduced in a *Δexl1* mutant strain of *P. atrosepticum*. As well as *Δexl1* exhibiting less maceration of potato plants, fewer bacteria are observed at distance from the inoculation site. However, bacteria infiltrated into the plant tissue are as virulent as the wild type, suggesting that this is due to alterations in the initial invasion of the tissue. Additionally, swarming from colonies grown on MacConkey soft agar was delayed in the mutant in comparison to the wild type. We found that Exl1 acts on the plant tissue, probably by remodelling of a cell wall component or altering the barrier properties of the cell wall inducing a plant defence response, which results in the production of ROS and the induction of marker genes of the JA, ET and SA signalling pathways in *Arabidopsis thaliana*. Exl1 inactive mutants fail to trigger such responses. This defence response is protective against *Pectobacterium brasiliense* and *Botrytis cinerea* in more than one plant species.

## Introduction

Expansins are non-hydrolytic proteins with cell wall-loosening activity in plants. They are encoded in all land plants where they have a cell wall modifying activity, important during different developmental processes^1^. In non-plant organisms, their biological role is not fully characterised but there is evidence that expansins are important for pathogenesis in some bacterial species^2–4^. Because of their structural similarities, it was initially assumed that plant and bacterial expansins had the same effect on the plant cell wall, however, *in vitro* extensibility experiments show higher levels of activity of plant expansins in comparison to their bacterial homologues. Expansins are composed of two tightly packed domains: domain 1 (D1) that resembles glycosyl hydrolase family-45 (albeit lacking certain catalytic residues), and for which conserved amino acids for loosening activity have been identified, e.g. Asp82 (in EXLX1 from *B. subtilis*) which is essential^5^; domain 2 (D2) is a family 63 carbohydrate binding module (CBM) according to the CAZY database with conserved aromatic amino acids for polysaccharide binding^1,5–8^. Active plant expansins belong to the EXPA (or α-expansins) family with high levels of creep activity for cell wall extension^9^. In contrast, *Zea mays* EXPB1, a representative of the EXPB (or β-expansins) family, acts on maize silk cell walls loosening and solubilising highly substituted glucuronoarabinoxylan, with the possible function of facilitating the pollen tube penetration into the maize stigma and style^10–13^. EXLA (expansin-like α) and EXLB (expansin-like β) are plant expansin-like proteins with no known function to date^9^. The EXLX family comprises all the expansin proteins from non-plant organisms, which seem to have evolved from the same ancestor as plant expansins^14,15^. Although EXLX1 from *B. subtilis* is a structural homologue of plant β-expansins, they display different activities^16^. The surface of bacterial expansin proteins is highly charged and, according to their net charge at pH 7, they are either basic (with theoretical pI > 9) or acidic (with theoretical pI < 6). This feature correlates with the type of organism in whose genomes they are encoded, whereby basic expansins are mainly found in Gram-positive bacteria, while genes encoding acidic expansins are mainly found in Gram-negative bacteria^17^. Expansin-containing microorganisms inhabit diverse ecological niches, but many of them interact with plant and algae material either as saprophytes, mutualists or pathogens, supporting the idea that the substrate for microbial expansins could also be part of the plant cell wall^15^. Accordingly, *Pectobacterium brasiliense*^18^ (formerly named *P. carotovorum* subsp. *brasiliense*) expansin-like protein 1 (Exl1), preferably binds to the xylem secondary cell wall in celery petioles, whereas *Bacillus subtilis* EXLX1 binds to the cell wall of distinct cell types^19^. The consequences of lacking expression of EXLX family members may be detrimental to bacterial interactions with plant hosts: *B. subtilis* expansin null mutants lose their ability to colonise maize roots by more than 90%^5^; infection symptoms in tomato are also reduced both for an expansin null mutant of *Ralstonia solanaceraum* and for *Clavibacter michiganensis* subsp. *michiganensis* carrying a truncated form of the plasmid-borne gene lacking the expansin module. Contrarily, the deletion of *C. michiganensis* chromosomic expansin (*ΔCmEXLX2*) increased disease symptoms in tomato^2,3^. Interestingly, of the very few bacteria (approximately 2% of all current annotated prokaryote genomes) with expansin genes, several are xylem-dwelling phytopathogens: *Pectobacterium*, *Ralstonia*, *Clavibacter*, *Xylella* and *Xanthomonas*, suggesting a role in adaptation to this plant structure. Consistent with this, expansin Exl1 from *P. brasiliense* binds a substrate surrounding the xylem vessels of celery^19^ and *Erwinia tracheiphila* expansin, along with a truncated GH5, are both necessary for proper infection through the xylem of squash^4^.

Members of the *Pectobacterium* genus are important expansin-possessing phytopathogens that cause economic losses worldwide and are found in the top ten plant pathogenic bacteria because of their economic impact and/or scientific importance^20^. *P. brasiliense* causes soft rot disease in crops and vegetables during cultivation, transportation and storage. It is widely distributed geographically and has a broad host range, including celery, broccoli, carrot, chard, beetroot, potato, tobacco and cactus^21^. On the other hand, *P. atrosepticum*, an inhabitant of temperate regions, is the main cause of blackleg disease in potato, to which it is almost exclusively restricted^20–22^. Despite these differences, both species share high similarity, with approximately 77% of the complete *P. atrosepticum* chromosome present in *P. brasiliense*^23^, and with some similarities in the pathogenesis-related control of gene expression. Both species are present in the soil and on plant surfaces, penetrating the tissues through host natural openings (such as lenticels and stomata), or are inoculated into wounds by chewing nematodes or insects, from which they move to the xylem structures by an unknown mechanism^21,22^. Within the tissue, and in a cell density-controlled manner, *Pectobacterium* species produce a variety of plant cell wall degrading enzymes (PCWDEs) including pectate lyases, cellulases, xylanases, polygalacturonases and proteases that are responsible for disease symptoms^24^.

PCWDEs secreted by pathogens release molecules derived from the polymers of the plant cell wall (such as oligogalacturonides^25^, cello-oligosaccharides^26^ or xyloglucan oligosaccharides^27^) named damage-associated molecular patterns (DAMPs) that trigger a plant defence response involving pattern recognition receptors (PRRs) and the induction of different signalling cascades^28–30^. The fungal expansin-related proteins cerato-platanin and swollenin are proteins without catalytic properties that also act on the plant cell wall triggering a plant defence response. Cerato-platanin’s single domain is similar to D1 of expansins and infiltration of this protein in tobacco leaves results in a systemic acquired resistance response protecting against infection by *B. cinerea* and *Pseudomonas syringae* pv*. tomato* after a subsequent challenge^31,32^. Swollenins are cellulose-acting proteins containing a CBM family 1 at the N-terminus and a C-terminal expansin-like domain. Previous infiltration of the swollenin CBM into cucumber leaves protects against infection with *B. cinerea* and *Pseudomonas syringae* pv *lachrymans*^33^. In the case of the nematodes *Globodera rostochiensis* and *Heterodera avenae*, it was also reported that the expansin-like proteins *Gr*EXPB2 and *Ha*EXPB2 respectively, could act as effectors inducing cell death in their plant hosts^34,35^. However, the mechanism by which these responses come about is not clear.

Here, we analyse the expression and regulation of expansin *exl1* in *P. brasiliense* BF45 and *P. atrosepticum* SCRI 1043. By altering the normal levels of *exl1* either by overexpression in *P. brasiliense* or in *P. atrosepticum*, or in a null mutant for *P. atrosepticum*, we identified Exl1 contribution to the infection of plant hosts. Our results suggest that Exl1 modifies the plant cell wall or its barrier properties, as the infiltration of pure preparations of wild type protein, but not of two inactive expansin mutants, triggers an immune response in *A. thaliana*. This response is consistent with the activation of a DAMP-mediated response, in which salicylic acid, ethylene and jasmonate pathway genes are induced. The Exl1-induced defence response protects *A. thaliana* against a subsequent challenge with *P. brasiliense* or *Botrytis cinerea* and is also observed in one other plant species (celery).

## Results

### *exl1* is expressed during plant infection and regulated by quorum sensing

**Figure 1:**
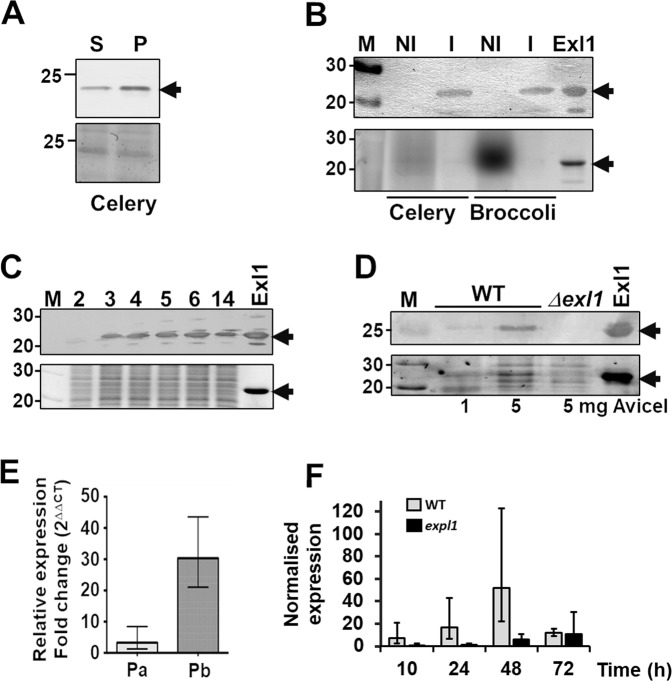
Expansin gene *exl1* is expressed during infection and is controlled by quorum sensing. (**A**) Western blot detection of Exl1 from infected celery (arrow, top panel), both in the supernatant (S) of the macerated tissue and in the insoluble fraction (P for pellet), in comparison to the protein loaded (bottom panel). (**B**) Anti-Exl1 antibody is specific for *P. brasiliense* Exl1, as it does not cross react with endogenous plant expansins (NI) *vs. P. brasiliense* BF45 infection (I). (**C)** Induction of Exl1 (arrow) through time is observed reaching a maximum at 6 h (≈1.3 OD_600_). (**D**) *P. atrosepticum* SCRI 1043 Exl1 expression in wild type and *Δexl1* strains grown in LB obtained by pull down with 1 mg and 5 mg of cellulose (Avicel); from B-D, top panel is the Western blot anti Exl1, and bottom panel is the loaded protein control containing 1 μg of pure Exl1 (arrow). **E**) Relative *exl1* gene expression level in *P. atrosepticum* SCRI 1043 (Pa) and *P. brasiliense* BF45 (Pb) infected potato leaves at 24 hpi. (**F**) Kinetics of relative *exl1* gene expression in *P. atrosepticum* wild type (grey bars) and a QS defective mutant (black bars) by RT-qPCR in infected potato leaves over a 72-h period. Error bars indicate+/− standard deviation of the average across three independent experiments (*n* = 24) (panels E and F). Complete Western blot membranes and protein gels are shown in Figs. S1 to S3.

Phenotypes for expansin gene mutants in phytopathogenic bacteria have been reported but analysis of expansin expression has not been addressed. We investigated the expansin Exl1 of *P. brasiliense* BF45 and *P. atrosepticum* SCRI 1043 (we gave both proteins the same name as they are orthologues, with 98.5% identity at the amino acid level). Firstly, we used *P. brasiliense* to analyse Exl1 expression during infection and in laboratory media. *P. brasiliense* infects several hosts, including celery and broccoli. Direct inoculation of the cells into celery petioles results in maceration after 24 h. Using an antibody against an epitope in D2, we detected Exl1 by western blot in both the soluble and insoluble fractions of the macerated tissue (Fig. 1A), but not in non-infected celery or broccoli (Fig. 1B), confirming the expression of *exl1* during infection by *P. brasiliense*. We then analysed for the presence of Exl1 protein in two conditions: (i) LB medium, in case *exl1* was regulated by population density; and (ii) in pectin-containing medium, in case pectin breakdown products were required^36^. Exl1 was observed both in the supernatant (after pulldown with Avicel having concentrated it 5-fold) and in the pellet of centrifuged bacteria (where it was easily detected) in both growing conditions, increasing in a cell-density dependent manner with a peak just before entering the stationary phase, which is suggestive of quorum sensing (QS)-regulation (Fig. 1C and Fig. S2). The level of Exl1 in *P. atrosepticum* was lower in comparison to *P. brasiliense*, thus to detect the protein by western blot from cells grown in LB or pectin media bacteria had to be lysed and Exl1 pulled down with insoluble cellulose from the supernatant of these preparations (Fig. 1D). Indeed, RT-qPCR showed 30-fold greater expression of *exl1* in *P. brasiliense* in comparison to *P. atrosepticum* (Fig. 1E). Subsequently, we used RT-qPCR to determine the induction of *exl1* during infection of potato leaves by *P. atrosepticum*. We observed a basal expression level of *exl1* during the first 10 h post infiltration followed by an increase with maximal expression levels after 48 hours (Fig. 1F). We confirmed that in *P. atrosepticum exl1* regulation is under the control of QS because *exl1* transcripts are down-regulated by approximately 8, 9 and 6-fold at 10, 24 and 48 post inoculation, respectively in the *expI* mutant, which is defective in the synthesis of N-(3-oxohexanoyl)-L-homoserine lactone (OHHL)^24^. However, other regulators might exist as a degree of expression was observed 72 h post-infiltration (Fig. 1F).

### Higher levels of Exl1 correlate with increased virulence towards the hosts

**Figure 2:**
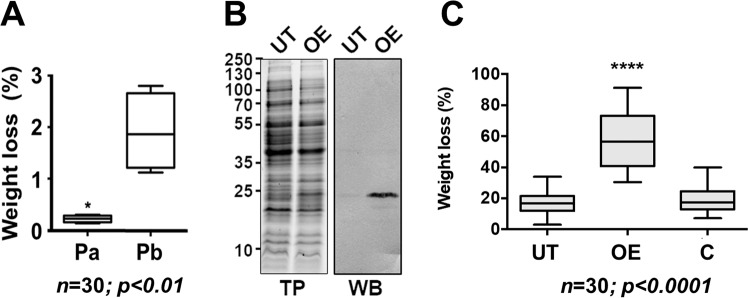
Expansin expression correlates with the level of infection. (**A**) *P. brasiliense* is more virulent than *P. atrosepticum* on potato tuber causing higher tissue maceration at 7 dpi/23 °C. Box plots (showing median and first and third quartiles and whiskers indicating the range of outliers) of three independent experiments (*n* = 30); significance *p* < 0.01 (*) was calculated with the Mann-Whitney test. (**B**) Western blot of the over-expression (OE) of *exl1* gene *P. brasiliense* BF45 compared to the untransformed strain (UT), and control of total protein (TP). Complete membranes with different contrasts can be found in the Supplementary materials. (**C**) Increased celery tissue maceration by *exl1* over-expression (OE) in *P. brasiliense* BF45 in comparison to untransformed *P. brasiliense* (UT) and to GFP overexpression as an irrelevant control (C). Box plots of three independent experiments (*n* = 30); sig*n*ificance *p* < 0.0001 (****) was calculated with Kruskall-Wallis tests for non-parametric data.

**Figure 3:**
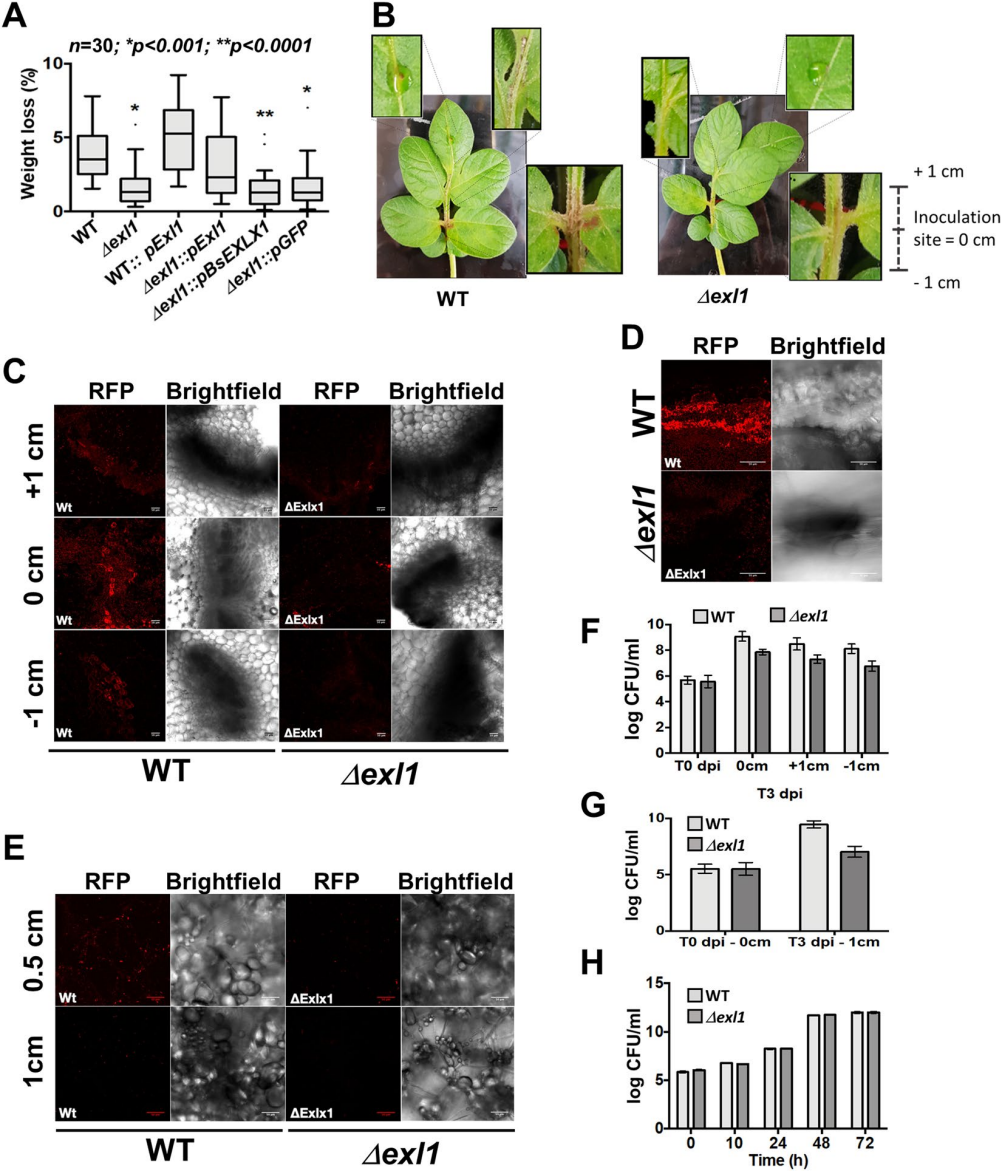
Expansin mutant of *P. atrosepticum*, *Δexl1*, has reduced infection symptoms. (**A**) Maceration levels of potato tubers is reduced in the expansin mutant in *P. atrosepticum* SCRI 1043 (*Δexl1*) compared to the wild type strain (WT) at 7 dpi/28 °C. Phenotype is rescued by over-expression of *exl1* gene reaching similar levels to the overexpression in the WT strain, but not by over expression of *B. subtilis EXLX1* or *GFP* irrelevant control. Box plots of three independent experiments (*n* = 30); significances *p* < 0.0001 (*) and *p* < 0.0001 (**) were calculated with Kruskall-Wallis tests for non-parametric data. (**B**) Early black leg infection symptoms induced in potato plants by inoculating *P. atrosepticum* WT or mutant *Δexl1* in a small wound into the petiole at the second nodule, or by placing a cell suspension drop on the surface of intact leaves, observed at 72 hpi. (**C**–**E)** Confocal images of samples obtained at the inoculation site (0 cm) or 1 cm above (+1 cm) or below (−1 cm) of potato petioles at 72 hpi with wild type (WT) or *Δexl1* cells expressing the red fluorescent protein (RFP) from petioles **(C)**; leaves **(D)**; and potato tubers at 0.5 cm and 1 cm from the potato surface **(E)**. (**F**–**H)** Colony count of bacteria recovered from: potato petioles at 0 cm on the inoculation time (T0), and at 3 dpi at +1 cm or -1 cm **(F)**; potato tubers at 0 cm, on the inoculation time (T0), and at 3 dpi at -1 cm **(G)**; fully infiltrated potato leaves at 3 dpi **(H)**. Error bars indicate the +/− standard deviation of the average of colony formation units (CFU) across three independent experiments (*n* = 6) (panels F-H).

We have determined that *P. brasiliense* produces a faster and greater extent of maceration (measured as tissue loss) in potato tubers compared to *P. atrosepticum* even at 23 °C (Fig. 2A), the temperature at which *P. atrosepticum* is most infective, which correlates with the level of expansin expressed by each species (Fig. 2A). We wished to determine whether modulating expansin expression could alter maceration of the host tissue. Due to difficulties in obtaining an expansin null mutant in *P. brasiliense*, the *exl1* gene was instead overexpressed in *P. brasiliense* under the control of the strong *TRP* promoter, achieving fifteen-fold greater expression in LB broth when compared to the wild type cells (Fig. 2B). This resulted in increased celery maceration (Fig. 2C). On the other hand, we created a null mutant of the expansin in *P. atrosepticum* (*Δexl1*) (Fig. 1D). Characterisation of this mutant showed that some phenotypes were indistinguishable from the wild type strain, including growth in LB and in minimal media containing pectin, the general activity of their PCWDEs (cellulases, pectinases and proteases), biofilm production and attachment to potato roots (Fig. S4). However, we observed a reduction of approximately three-fold tissue maceration in potato tubers with respect to the wild type strain (Fig. 3A). Inoculation of wounded potato petioles with the mutant strain also caused fewer symptoms than the wild type strain, which additionally showed tissue damage away from the inoculation site (Fig. 3B). When bacteria, either wild type or mutant, were allowed to enter the tissue through the natural openings of the plant (i.e. not creating a wound in the tissue), by placing a cell suspension drop on intact potato leaves, a dark mark appeared three days later in the case of infection with wild type cells but not with the mutant (Fig. 3B). The low maceration phenotype of *Δexl1* was reversed and slightly increased by over-expression of *exl1*, similar to the wild type strain when also overexpressing *exl1* (Fig. 3A). We then asked whether *B. subtilis* EXLX1 (a basic expansin) could also complement the *Δexl1* strain using a plasmid with the *BsEXLX1* gene under the control of the *TRP* promoter. Surprisingly, this construct was unable to recover the infectivity defect of the mutant (Fig. 3A), suggesting that either Exl1 and *Bs*EXLX1 have different activities or targets on the cell wall, possibly due to the relative difference in their electric charge. Another possible explanation would be that *Bs*EXLX1 was not properly folded in our *Pectobacterium* strains. Finally, as expected, overexpressing an irrelevant gene (*GFP*) did not rescue the mutant phenotype (Fig. 3A).

To further characterise the differences between the infectivity of *P. atrosepticum* wild type and *Δex1*, we transformed both strains with a plasmid expressing the red fluorescent protein and observed the cells by confocal microscopy three days after inoculation in potato plants. We prepared cross-sections of the inoculation site (which corresponded to the second petiole node -designated as 0 cm) and from the tissue one centimetre above (+1 cm) and below (−1 cm) this site (Fig. 3B). The fluorescence was more intense in samples infected with the wild type strain at all locations (0, +1 and −1 cm) in comparison with the mutant (Fig. 3C) but in both cases the signal was weaker at −1 cm, in comparison to +1 cm, probably because cells tended to move to the upper part of the plant (Fig. 3C). When cells entered the leaves via the natural plant openings there was also a stronger fluorescent signal in the case of the wild type strain (Fig. 3D). Comparable results were obtained with inoculated potato tubers (Fig. 3E). The difference in fluorescence between wild type and mutant could be attributed to at least two factors: firstly, that the mutant cells are more susceptible to the plant defences, although measuring *in vitro *cell survival of the strains under stress in the presence of oxidative species (H_2_O_2_) or in hyperosmotic medium did not indicate any difference (Fig. S5); secondly, that the same number of cells were present but that the mutants lost the plasmid faster than the wild type strain. Cell counts (CFUs) at three-days post infection confirmed that there were indeed fewer cells in the *Δexl1* mutant than the wild type strain, including at the inoculation site after starting the experiment with equal numbers of cells, both in the petioles (Fig. 3F) and in tuber (Fig. 3G). In contrast, when mutant and wild type strains were infiltrated into the tissue of potato leaves under vacuum, we observed no difference in the number of bacteria counted for each strain, nor were there differences in the degree of maceration (Fig. 3H). This suggests that the virulence of *Δexl1* cells is comparable to wild type *P. atrosepticum*, but that the mechanism by which the bacteria access the plant tissue is somehow affected.

### Expression of *exl1* correlates with the swarming motility in *Pectobacterium atrosepticum*

**Figure 4:**
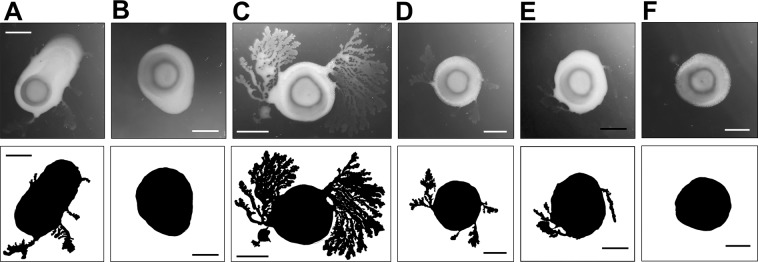
Exl1 is required for swarming motility in *P. atrosepticum*. Colonies, (and colonies silhouette at the bottom) growing in 0.4x MacConkey agar 48 h/28 °C, of: **(A)** *P. atrosepticum* wild type; **(B)** *Δexl1* strain; **(C)** wild type *P. atrosepticum* over-expressing *exl1*; **(D)** *Δexl1* strain over-expressing *exl1*; **(E)** *Δexl1* strain over-expressing *BsEXLX1*; **(F)** *Δexl1* strains over-expressing *GFP* as a control. Bar = 295 pixels.

We analysed whether the expansin mutant could have a motility defect. Indeed, mutant cells grown in soft MacConkey agar showed delayed swarming in comparison to the wild type, which under the same conditions managed to extend colony projections or tendrils composed of elongated and hyper-motile cells. The mutant strain eventually produced tendrils (approximately 24 h later than the wild type strain) but always in lesser quantities than the wild type, which in turn showed larger and more abundant tendril that grew faster when overexprssing exl1 gene (compare Fig. 4A to Fig. 4C). On the contrary, *Δexl1* cells swam similarly to wild type cells (Fig. S6), which indicates that their flagella are normal. The swarming defect was recovered by complementing with both *exl1* and *BsEXLX1* genes (Fig. 4A–F), although at this point we are unable to correlate this plant-independent phenotype with the infectivity of the strains.

### Infiltrated Exl1 affects the plant cell wall and protects against pathogens in *Apium graveolens* and *Arabidopsis thaliana*

**Figure 5:**
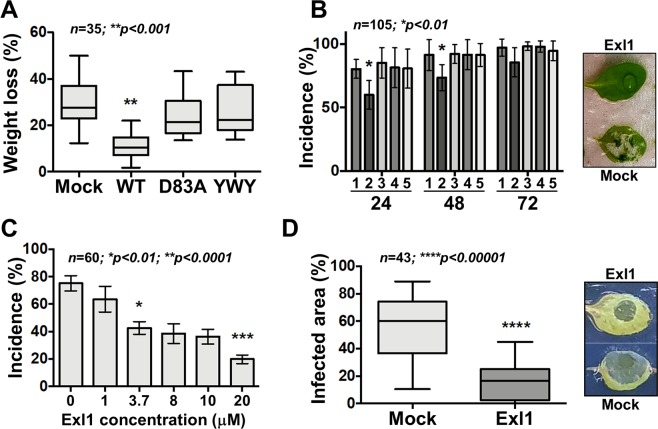
Exl1 activity protects from pathogens challenge in different hosts. (**A**) Maceration levels of celery petioles challenged with *P. brasiliense* BF45, 24 h after previous infiltration with buffer (Mock), or 3.7 μM pure Exl1 (WT), or expansin inactive D83A, and Y125A/W126A/Y157A (YWY). Box plots of three independent experiments (*n* = 35); significance *p* < 0.001 (**) was calculated with Kruskall-Wallis tests for non-parametric data. (**B**) Likewise, incidence of *Arabidopsis thaliana* leaf maceration levels challenged with *P. brasiliense* BF45, 24 h after previous infiltration with buffer (#1 bars), or 3.7 μM pure Exl1 (#2 bars), *B. subtilis* EXLX1 (#3 bars), D83A mutant (#4 bars), and YWY (#5 bars), over a 72-h period. An example of protected (Exl1) or macerated (Mock) leaves is shown on the right. Error bars indicate the +/− standard deviation of the average across at least three independent experiments (*n* = 105); sig*n*ificance *p* < 0.01 (*) was calculated according to a Kruskall-Wallis test for non-parametric data. (**C)** Dose-dependent protection conferred by infiltrated Exl1 in *A. thaliana* leaves treated 24 h previously, challenged with *P. brasiliense* BF45. Error bars indicate the +/− standard deviation of the average across at least three independent experiments (*n* = 60); significances *p* < 0.01 (*) and *p* < 0.0001 (***) were calculated according to a Kruskall-Wallis test for non-parametric data. (**D)** Exl1 protection to infection 24 h after previous incubation with the pathogenic fungus *Botrytis cinerea*. An example of protected (Exl1) or infected (Mock) leaves is shown on the right. Box plots of at least three independent experiments (*n* = 43); significance *p* < 0.00001 (****) was calculated according to a Mann-Whitney test for non-parametric data.

We have previously reported that Exl1 binds to the secondary cell wall of xylem vessels and that incubation of isolated helical bundles with Exl1 releases an unidentified polysaccharide^19^. Thus, we asked whether Exl1 could also be modifying the cell wall *in planta*. For this, we infiltrated pure Exl1 protein in celery petioles, then challenged with *P. brasiliense* 24 h after infiltration and observed reduced maceration in comparison to the infiltration of the buffer (mock) (Fig. 5A). We repeated the experiment with the Exl1 activity-dead mutants D83A in D1 and the triple aromatic Y125 A/W126A/Y157A mutant (YWY) in D2, followed by a challenge with bacteria, after which we observed the same level of maceration as in the buffer control (Fig. 5A). These results suggest that Exl1 activity, rather than the protein, is being detected by the plant, probably activating a defence response. To further analyse this protective phenotype, we switched to using *Arabidopsis thaliana* as more powerful genetic tools exist for this species and it is also susceptible to *P. brasiliense* infection. Once more, we observed less maceration with Exl1 pre-infiltration followed by challenge with *P. brasiliense* (Fig. 5B), which was also dependent on the expansin activity as the inactive mutants failed to stimulate the protective response. Again, *B. subtilis* EXLX1 infiltration was unable to protect against infection with *P. brasiliense*. Exl1 protection was still significant after 48 h of Exl1 infiltration and evident after 72 h, when the pathogen population is large enough to surpass the plant immune system (Fig. 5B). The incidence of macerated *A. thaliana* leaves was dependent on the amount of infiltrated Exl1, which we considered an indirect measurement of the protein activity (Fig. 5C). Exl1 activity on *A. thaliana* leaves also reduced the symptoms of *B. cinerea* infection (Fig. 5D), indicating the onset of a general plant defence response against necrotrophs.

### Exl1 triggers a ROS burst and plant defence responses dependent on JA and SA

**Figure 6:**
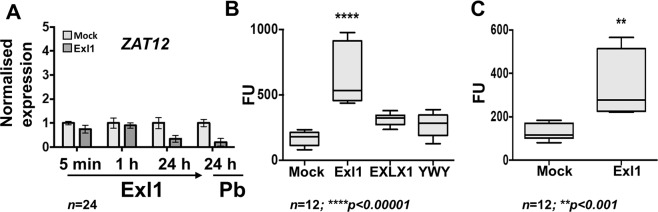
Exl1 infiltration triggers a burst of reactive oxygen species. (**A**) Relative expression level of ROS marker gene *ZAT12* by RT-qPCR. (**B**,**C)** Reactive oxygen species detected with dichloro-dihydro-fluorescein diacetate (DCFH-DA) in *A. thaliana* leaves 30 min **(B)** and 90 minutes **(C)** post infiltration with the indicated buffer (Mock), or 3.7 μM proteins: Exl1, *B. subtilis* EXLX1 and the Exl1 triple mutant Y125A/W126A/Y157A (YWY). Fluorescent areas were determined using the Fiji software; FU are fluorescence units in pixels/cm^2^. Box plots of at least three independent experiments (*n* = 12); significances *p* < 0.001 (**) and *p* < 0.00001 (****) were calculated according to an ordinary one-way ANOVA.

Protection towards pathogens is the result of a priming event, in this case the presence of Exl1, due to the accumulation of ROS and the induction of defence pathways mediated by hormones such as salicylic acid (SA), jasmonic acid (JA) and ethylene (ET). We determined whether marker genes of these pathways were triggered by Exl1 in *A. thaliana* leaves using RT-qPCR analysis at the following timepoints: 5 minutes, 1 h and 24 h post infiltration with Exl1 only. We also performed the same analysis after a challenge of 24 h with *P. brasiliense*. ROS accumulation is among indicators of activation of plant defences and we thus evaluated the expression of *ZAT12*, a marker gene for ROS. No induction of *ZAT12* was observed at any of the timepoints analysed and we even observed a 3.3-fold reduction at 24 h and again (five-fold change) 24 h after infection with *P. brasiliense* (Fig. 6A). Because ROS accumulate in a pulsating fashion^37^, to determine their presence we used the fluorescent dye dichloro-dihydro-fluorescein diacetate (DCFH-DA) and found an increased signal at 30 min (Fig. 6B). This increase was still detectable 90 min post treatment with Exl1 (Fig. 6C), indicative of a wave-like dynamic in the production of ROS.

**Figure 7:**
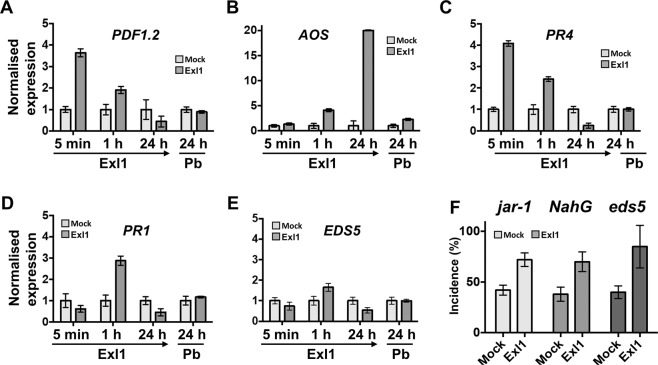
Exl1 activity triggers a plant defence response involving JA, ET and SA pathways in *A. thaliana* leaves. Normalised RNA expression levels by RT-qPCR of marker genes involved in the biosynthesis of plant defence hormones: jasmonic acid, *PDF1.2* **(A)** and *AOS* **(B)**; ethylene, PR4 **(C)**; salicylic acid, *PR1* **(D)** and *EDS5* **(E)**. Expression was analysed 5 min, 1 h and 24 h after buffer (Mock) or 3.7 μM Exl1 infiltration, and again 24 h after a challenge with *P. brasiliense* (Pb) previously treated with Exl1 for 24 hours. **(F)** *A. thaliana* mutants impaired in the synthesis of JA (*jar-1*) and SA (*NahG* and *eds5*) previously treated (for 24 h) with Exl1 fail to confer protection and become more susceptible towards *P. brasiliense* BF45. Error bars indicate the +/− standard deviations of the average across three independent experiments (*n* = 70); no significant differences were determined with a Mann-Whitney test for non-parametric data.

Then, we analysed marker genes for the JA, SA and ET pathways after infiltration with Exl1. The *PDF1.2* (a JA-induced plant defensin) was up-regulated by a 3.6-fold and 1.9-fold change during the early immune response after 5 min and one hour of treatment, respectively, but repressed by 2.2-fold at 24 h post Exl1 treatment (Fig. 7A). *ALLENE OXIDE SYNTHASE* gene (*AOS-* a biosynthetic JA gene) was 1.3-fold and 20-fold up-regulated at 1 h and 24 h, respectively, after infiltration of Exl1 (Fig. 7B). To monitor the ET-induced response, we analysed gene *PR4* that showed a 4-fold induction after 5 min and 2.4-fold induction at 1 h post Exl1 treatment, but it was 5-fold down-regulated after 24 h, showing a similar expression pattern to *PDF1.2* and in agreement with the synergism reported for ET and JA pathways^38^ (Fig. 7C). The SA-responsive genes *PR1* and *EDS5* were 2.8 and 1.6-fold induced after 1 h but down-regulated at 24 h, respectively (Fig. 7D,E). These data indicate that SA-, JA- and ET-elicited responses are modulated at different times by the exogenous application of Exl1. To further confirm the involvement of JA and SA pathways, we used the *jar-1* and *eds5 A. thaliana* mutants, impaired in the JA^39^ and SA^40^ pathways respectively, and also the transgenic *A. thaliana* line *NahG* which is deficient in SA accumulation^41^. In all cases, mutants treated with Exl1 lost the protective effect towards *P. carotovorum* BF45 infection, in contrast to the wild type *A. thaliana* (Fig. 7F). These data confirmed that the JA and SA pathways are involved in the immune response triggered by Exl1.

## Discussion

Here, we have shown that the expansin Exl1 protein is produced during infection by *P. brasiliense* and *P. atrosepticum*; that higher levels of Exl1 correlates with a more virulent phenotype and, conversely, absence of expansin produces less severe symptoms in potato. Thus, Exl1 can be considered as a virulence factor, which along with other pathogenesis-related genes (including those for the degradation of the plant cell wall), seems to be under QS-regulation. Previous work on QS-controlled genes important for pathogenesis in *P. atrosepticum* and *P. carotovorum* consistently identified gene ECA2220, annotated as a putative endocellulase^42,43^, which corresponds to the *exl1* ortholog of *P. brasiliense*. ECA2220 and Exl1 differ in three residues in the mature protein (considering Gln24 in the complete sequence as Gln1 after the predicted site of the secretion signal peptide): S41N, D147N, and V151I in ECA2220 compared to Exl1. It was determined that ECA2220 is an acidic protein (pI of approximately 4) that is secreted through the TSS2 system in a similar fashion to the PCWDEs CelV, PelC and PrtW^42^. Similarly to our findings, in *P. carotovorum* (*E. carotovora* subsp. *carotovora* strain MS20) ECA2220 expression is induced two-fold on addition of the quorum sensing autoinducer N-(3-oxo-hexanoyl)-L-homoserine lactone (OHHL) but its expression was not entirely dependent on OHHL, since some basal expression was observed in a mutant *carI*^−^ (*expI*) strain, unable to synthesize OHHL^24^. In the same work, authors determined a 7.5 and 6.9-fold reduction of ECA2220 transcript by RT-qPCR at 12 and 20 h post-inoculation, respectively, in an *expI* mutant compared to the wild type *P. atrosepticum*, similarly to our results.

We found Exl1 protein in the supernatant of macerated tissue in celery, broccoli and potato following infection by *P. brasiliense* but, additionally, Exl1 was also found in the insoluble fraction containing digested plant material and bacterial cells, to which it must be bound. Exl1 binds cellulose and possibly other polysaccharides and, given that in LB medium (i.e. lacking plant cell walls) Exl1 is abundant in the pellet of non-lysed cells suggests that it could also bind exopolysaccharides (EPS) from the cell wall of *Pectobacterium*. Similarly, *B. subtilis* EXLX1 readily binds peptidoglycan^5^; however, the possibility of EXLX1 binding to the bacterial cells needs to be analysed, together with the effect this could have on the interaction with the plant roots given the drastic reduction in colonization of the expansin mutant strain. A possible effect of Exl1 binding to its own EPS would result in alterations to the amount produced or the structure of the EPS, but we observed that in the absence of Exl1 the quantity of biofilm in *P. atrosepticum* was not affected. However, quantification cannot discriminate the integrity of the biofilm between the mutant and the wild type if it existed. Our expansin mutant showed reduced disease symptoms and reduced cell counts in potato compared to the wild type control after wound inoculation, but this was not due to an increased sensitivity of the mutant to the plant defences because, when infiltrated directly into potato leaves, both mutant and wild type grew at comparable rates. Additionally, when challenged with oxidative or hyperosmotic stress *in vitro* the mutant strain was as resistant as the wild type (Fig. S5). Thus, we interpreted the low number of recovered mutant bacteria as a defect of this strain for colonization compared to the wild type, probably due to a reduced tissue penetration. Interestingly, when β-expansin activity is reduced (in a transposon insertion mutant in gene *EXBP1* or by RNA silencing in maize), a decreased efficiency of pollen tube penetration into the stigma and style tissue was also determined^13,44^. EXPB1 activity includes solubilisation of key polysaccharides from the middle lamella that cement neighbour cells; these polysaccharides can then be detected in the supernatant of reactions containing deproteinised walls^11,12^. We have previously reported an *in vitro* polysaccharide solubilising activity for Exl1 from vascular bundles isolated from Swiss chard^19^, resembling the effects of EXPB1 on maize silk, which could explain our observations in case the same effect of Exl1 occurred *in planta*. We hypothesise that Exl1 could be acting on the potato cell wall to allow cells to move through the plant tissue, because although symptoms are reduced, maceration of the tissue ensues given the normal expression of other PCWDEs. In support to this invasion/penetration phenotype of the expansin mutant, Rocha, *et al*.^4^ recently observed that an expansin and GH5 double mutant in *E. tracheiphila* is impaired in colonising sites distant to the inoculation point, and attributed this phenotype to a bacteria movement defect. Interestingly, the mutant *ΔEXLX1* of *Ralstonia solanacearum* also shows wild-type virulence when directly introduced into the tomato vasculature, in contrast to inoculation in the soil where a less virulent phenotype was observed. This supports a requirement for expansins for optimal access into the host, although in this case it was due to hyper-attachment of the bacteria to tomato roots rendering them unable to enter the plant vasculature^3^. Other phenotypes have been found for expansin mutants in different bacteria, but it is not clear if accessibility to the host is involved. The expansin deletion mutant of *B. subtilis* for instance, shows very low colonization ability of maize roots but the underlying cause has not been analysed^5^. In contrast, our findings and others show that root attachment in *P. atrosepticum* and *C. michiganensis*^3^ was unaffected in the respective expansin mutants, indicating that expansin function is not involved in root attachment in these species.

We dismissed the possibility that the defect of our expansin mutant was due to flagellar defects, given that its swimming capacity was comparable to the wild type strain (Fig. S6), but surprisingly we found a difference in swarming on MacConkey soft agar. Swarming motility is an energetically costly displacement towards more nutrient rich sites, which includes cell differentiation of the swarmer cells that become elongated and hypermotile in comparison to non-swarmer cells^45,46^. In different species, swarming correlates with virulence^47,48^. We have determined that Pectobacterium swarmer cells are elongated and hypermotile^49^, which are characteristics of a proportion of cells that we have found in the macerated tissue of infected vegetables, probably belonging to the population that travels away of the symptomatic site to invade other parts of the host. At this point we can only speculate on the role of Exl1 during swarming but it is possible that part of the exported protein remains attached to the EPS conferring electrostatic properties to the bacteria’s surface that are important for cell movement on a hydrated matrix. Interestingly, a transposon insertion mutant in gene EXPB1 of maize pollen has an aggregative phenotype and a dehydration defect that the authors attribute to altered hydrodynamic properties of the pollen coat or wall due to a lesser abundance of expansin on the pollen surface^13^. If *Pectobacterium* EPS is a target for Exl1, this could influence swarming and pathogenesis.

Our data show that Exl1 activity on the cell wall, and not the protein *per se*, stimulates a defence response reminiscent to the induced by damage associated molecular patterns (DAMPs)^30,50^ or by wall integrity alterations, such as reduction of cellulose biosynthesis^51,52^. Both mechanisms involve JA, ET and SA pathways, ROS increase and resistance to pathogens. Indeed, Exl1 activity triggered a plant defence response resulting in resistance towards bacterial and fungal pathogens. We found induction of marker genes of the SA, JA and ET pathways and the protection provided by Exl1 was lost in JA- and SA-related mutants. Because the SA pathway is fundamental for acquisition of induced systemic resistance (ISR) in plants^53^, we can hypothesize that Exl1 could also induce ISR in plants.

Our data cannot inform on the chemical nature of the elicitor molecule, but there is some evidence to support pectin involvement in Exl1 activity: i) our previous results of Exl1 polysaccharide-releasing capacity from the xylem of Swiss chard; ii) Exl1 preference for binding the intercellular spaces of the xylem vessels and the surrounding cells, which are enriched in pectin; iii) a negative surface electric charge of Exl1 that could serve to repel and dissociate negatively charged pectin from cellulose; iv) production of ROS with pectin derivatives elicitation, but not with cello-oligosaccharides or xyloglucan oligosaccharides. There is, however, a limitation to this hypothesis regarding the size of the elicitor. Defence elicitation with pectic derivatives (oligogalacturonides -OGAs) depends on their polymerisation degree (DP) being 10–14 optimal. Larger DPs would be restricted to reach the plasma membrane by the cell wall but elicitation occurs in protoplasts or isolated membranes with large fragments^54^. We hypothesize that if pectin was solubilised by Exl1 it could become the target of native plant polygalacturonases that would generate oligogalacturonides and trigger the defence response, assuming the presence of active pectin degradative enzymes at the time of Exl1 incubation. However, this might not be the case in our experiments given that in *A. thaliana* pectinases are found during leaf expansion^55^, which does not correspond to the timing of Exl1 treatment, nor the treatment of celery petioles. Otherwise large detached fragments would need to be close enough to the membrane receptors to directly activate the response. Alternatively, Exl1 activity could be altering the barrier properties of the cell wall due to polysaccharides creep, as it has been shown for some bacterial and fungal expansins^56^. Although creep levels by microbial expansins seem marginal in comparison to plant expansins, this might be sufficient for their biological function. Indeed, microcrystalline cellulose creep and reduced tensile stress of filter paper were demonstrated by Lior, *et al*., 2016 for expansin *Ccl*Exl1 (as part of cellulosome fractions) from *Clostridium clariflavum*, even though *Ccl*Exl1 is expressed at low levels^57^. Perturbations to the cell wall rigidity are sensed by a family of kinase receptors (malectine-like receptor kinases) on the cell membrane^58^, such as FERONIA (FER) that additionally positively regulates pattern triggered immunity^59^, and THESEUS1 (THE1) that participates on the response to cell wall damage during inhibition of cellulose biosyntheis^60^. The ligand for THE1 upon cellulose synthesis inhibition remains obscure, although some authors speculate that it could be the cellulose synthetic complex itself or possibly a detached molecule. In the case that Exl1 provoked an alteration to the cell wall integrity, the plant response dependent on malectine-like receptor kinases could explain the observed reduced symptoms of the hosts after Exl1 treatment and a posterior pathogen challenge, since this pathway mediates ectopic lignin deposition. Still, the identity of the ligand for the receptor persists under this scenario. Finally, differently to our observations the *A. thaliana* mutant, *jar-1*, deposits more lignin than the wild type after treatment with cellulose synthase inhibitor isoxaben^51,52^, whereas the same mutation became more sensible in our system.

Other microbial expansin-like proteins have shown immune responses in plants, but in contrast to the presence of cerato platanins (CP) from fungi^61^ or the nematode expansins *Gr*EXPB2^34^ and *Ha*EXPB2^35^ that cause plant necrosis (due to the hypersensitive response) Exl1 activity failed to produce cell death, in agreement with elicitation of pattern triggered immunity. This suggests that despite the similarities of these proteins they act in different ways and/or have different targets in the plant cell wall. Furthermore, we found that despite their predicted similar structure and conserved polysaccharide binding surface, Exl1 and *Bs*EXLX1 could have different roles or even different mechanisms of action, as we have not found a solubilising function for *B. subtilis*^19^ and it failed to prime the plant against *P. brasiliense* in our experimental conditions. Cell wall binding patterns between Exl1 and *Bs*EXLX1 differ due to distinct electric superficial charge, in which the unproductive binding through electrostatic interactions with the negative cell wall components dominates over the polysaccharide binding surface in *Bs*EXLX1. This is reversed by mutation of the positive residues R173Q/K180Q/K183Q (RKK), which increases the productive binding of *Bs*EXLX1 onto cell wall microdomains^62^. These differences clearly provide an evolutionary advantage to different bacteria for successfully interacting with their host and it is possible that their structural similarities are unrelated from a biological point of view. Future work would tell whether *Bs*EXLX1 mutant RKK elicits a plant response and whether this affects the interaction of *B. subtilis* with its host, as the reason for the drastic difference in charge among bacterial expansins is yet unknown.

## Conclusion

Due to of the lack of hydrolytic catalysis of bacterial expansins their molecular mechanism has been elusive. By learning from the effects that they produce on plants, we may better understand their activity. Our results suggest that expansin Exl1 from *Pectobacterium* is a virulence factor that might be acting in a similar manner to 𝛽-expansins during the infection process, by facilitating bacterial movement through the plant tissue to reach the xylem. This activity makes Exl1 a novel protein effector that triggers an immune response dependent on salicylic acid, jasmonic acid, ethylene and ROS pathways. Furthermore, Exl1 induces resistance towards important bacterial and fungi phytopathogens, opening the possibility to explore its potential as a biocontrol molecule.

## Material and methods

### Media and strains

All strains were cultured on Luria Bertani (LB) agar or broth (with antibiotics when necessary) at 28 ± 2 °C with aeration. For some experiments Liquid Enrichment Medium (LEM_AG366_) composed of 1 mM NaOH, 1.5 mM MgSO_4_, 7.5 mM (NH_4_)_2_SO_4_, 5.7 mM K_2_HPO_4_ and 0.17% AG366 pectin from Agdia, Biofords, was used. *P. brasiliense* strain BF45 was kindly donated by Dr Oscar Mascorro from Universidad de Chapingo. *P. atrosepticum* SCRI 1043 and *expI* mutant have been previously described^24^. To create the expansin null mutant *Δexl1*, ~600 bp of the upstream and downstream regions of the expansin gene were PCR-amplified using the primers *Xba*I-fw, *Hind*III-rev, *Xho*I-fw and *Apa*I-rev described in Supplementary Table 1. To generate the marker exchange plasmid, this fragment lacking the expansin gene or a resistance gene cassette was cloned into the suicide vector pKNG101^63^. The plasmid was introduced into *P. atrosepticum* SCRI 1043 by conjugation. The disruption of the gene was confirmed by PCR analysis, DNA sequencing and western blot.

### Infection assays

Celery, broccoli, potato (leaves and tubers) were disinfected with 5% sodium hypochlorite and washed with distilled water. For the inoculation of celery and potato petioles a cut (~5 mm) was opened with a cutter and inoculated with 20 μl of a bacterial dilution adjusted to 0.1 OD_600_ (10^8^ cells). A drop containing ~10^9^ cells was placed on the upper surface of potato leaves. Tubers were stabbed into the surface using a yellow pipette tip and inoculated with 10 μl of ~10^8^ cells, and the orifice was sealed with Vaseline. Experiments were incubated at room temperature (RT) for three days in a humidity chamber. Plants were weighed before and after the macerated tissue was removed. Experiments were performed at least three times with sample numbers indicated in the corresponding figure legend (*n*). For data with non-Gaussian distributions (determined with a Kolmogorov-Smirnov normality test), statistical analyses were calculated according to the Mann-Whitney test (when comparing two samples) or the Kruskall-Wallis test for group comparison of non-parametric data, using GraphPad Prism version 6.04, GraphPad Software (La Jolla California USA).

### Protein preparation for Western blot

Macerated tissue from infection with *P. brasiliense* BF45 was scooped out with a spatula and placed in clean 1 ml tubes, then centrifuged in a microcentrifuge at maximum speed at 4 °C. Protein concentration was determined in the supernatants with Protein Assay Dye Reagent Concentrate (BioRad) following the manufacturer’s instructions. Six micrograms were run in SDS-PAGE. Pellets were resuspended in one volume of SDS-sample buffer and after boiling, 20 μl were run in SDS-PAGE. Macerated tissue from potato tubers infected with *P. atrosepticum* wild type SCRI 1043 or *Δexl1* strains, was collected and clarified by centrifugation and proteins quantified as indicated above, then 500 μl of 5-fold concentrated supernatant (using Vivaspin filters with 10 kDa cutoff) containing 2.5 mg of protein were incubated with 5 mg Avicel (Sigma) 1 h/30 °C/1000 rpm, then centrifuged at maximum speed in a microcentrifuge, pellets were washed three times with 1x PBS. Proteins bound to Avicel were solubilised in 20 μl of SDS-sample buffer and run in 10% SDS-PAGE. For Exl1 level analyses through the growth curve: cell cultures of *P. brasiliense* BF45 in LB or LEM_AG366_ media were initiated at 0.04 OD_600_. One ml samples were taken every hour for the indicated times. Samples were centrifuged and pellets were resuspended sterile water volumes (μl) according to a 130 relation volume:OD_600_, then vortexed at maximum speed for 30 seconds at 4 °C and centrifuged again. Finally, 15 μl of the supernatant were mixed with SDS-sample buffer, boiled and run in 10% SDS-PAGE. Five micrograms of protein from non-infected celery and broccoli were obtain after mechanical maceration of disinfected frozen tissue (using liquid nitrogen) in a mortar. Gels were incubated 1 min in 5% 2,2,2-trichloroethanol and visualised in a Gel Doc EZ Imager (BioRad) using the Stain-free tray.

### Western blot

A polyclonal antibody developed in rabbit against the chemically synthesized peptide GMNDIPIEFTDVKG corresponding amino acids 167 to 180 of domain 2 of Exl1 was purchased from GeneScript. After SDS-PAGE, proteins were transferred to PVDF membranes (Millipore) and blocked with 5% skimmed milk. Anti-Exl1 primary antibody (diluted 1:40,000 in 1x PBS – 0.05% Tween 20) and secondary antibody (rat anti-rabbit-HRP 1:3000 -Invitrogen) were incubated 90 min each at room temperature with gentle shaking, followed by three washes with PBST, 5 min each after antibody incubation. Bands were developed with Novex HRP Chromogenic Substrate (TMB) (Thermo Scientific).

### Quantitative RT-PCR

***exl1 expression****.* Potato leaves (4 per replica) were infiltrated with a bacterial suspension of *P. atrosepticum* wild type, *Δexl1*, or *ΔexpI (*10^7^ cells/ml) using a vacuum pump and 0.8 bar for 10 min. Infiltrated leaves were placed in plastic boxes with moist paper and incubated for 72 h at 22 °C. Samples were harvested at 0, 10, 24, 48 and 72 h and processed to determine the total number of bacterial cells (see below) or flash-frozen in liquid nitrogen for RNA extraction. Total RNA was isolated using the RNeasy Plant mini kit (Qiagen). Contaminant genomic DNA was digested in RNase-free DNase columns (Promega). cDNA was synthesized with 1 μg of RNA using SuperScript III First Strand Synthesis System (Invitrogen). Primers for RT-qPCR are listed in Supplementary Table 1. PCR reactions were performed in a final volume of 12.5 μl containing: 6.25 μl of Sybr Green Master Mix 2×, 0.3 μM of each primer, 1 μl of diluted cDNA template (1:10) and 3.75 μl of HPLC water. PCR conditions were 95 °C for 10 min followed by 40 cycles of 95 °C for 15 s and 60 °C for 1 min. Controls without template were included for each primer pair. Expression of gene *recA* was used as a reference.

### *A. thaliana* immune response genes expression

Quantitative RT-PCR of defence marker genes for *A. thaliana* was performed as follows: leaves (8 per replica) were frozen in liquid nitrogen and ground at the following times after Exl1 infiltration: 5 min, 1 h and 24 h; and 24 h following a challenge with *P. brasiliense* BF45. Total RNA was extracted using TRIzol reagent (Invitrogen). One μg of RNA was used for the synthesis of cDNA using oligo dT and ProtoScript First Strand cDNA Synthesis Kit (New England Biolabs). PCR reactions contained 1 μl of cDNA (diluted 1/40) in Maxima SYBR Green/ROX qPCR Master Mix (2×) (ThermoFisher Scientific) and 0.5 μm of specific primers (described in Supplementary Table 1). PCR conditions were: 95 °C initial denaturation for 15 min, 45 cycles of 15 s/95 °C, 30 s/60 °C and 30 s/72 °C. Expression of gene At4g26410 was used as reference for normalization^64^. All reactions were performed in 96-well plates using the Applied Biosystems StepOne and StepOnePlus Real-Time PCR System (ThermoFisher Scientific). Normalized gene expression was determined using the comparative 2^−ΔΔ*CT*^ method previously described^65^.

### Mutant complementation and *exl1* over-expression

Expansin gene was PCR-amplified from genomic DNA of *P. brasiliense* BF45 with primers XhoI-fw and SalI-rev (described in Supplementary Table 1) and cloned into pTB93F vector (kindly donated by Professor Sharon R. Long) between *Sal*I and *Xho*I sites, and under the signal peptide with sequence MRRWRALLLAASVAVAPGLPATAA. Plasmids pTB93F-Exl1 and pTB93F (that express GFP as a control) were transformed by electroporation into BF45 strain competent cells. Briefly, cells were grown at 28 °C to mid-log phase between 0.7 and 0.8 OD_600_ then chilled for 20 min on ice, and pelleted for 15 min/4000x g, washed 2x in 10% ice-cold glycerol and resuspended in 1/20 of the original culture volumes with 10% glycerol. Cells were electroporated in 0.1 cm cuvettes with 10 ng of plasmid by a pulse of 1.8 kV (Bio Rad). Cells were recovered in SOC medium for 1 h/28 °C/200 rpm shaking. Transformants were selected with 100 μg/ml spectinomycin and 100 μg/ml chloramphenicol. Exl1 levels were analysed in 5-fold concentrated supernatant of cultures grown at 28 °C overnight in LEM_AG366_ containing antibiotics by pull-down with 5 mg Avicel (Sigma), and subsequent SDS-PAGE with 50 μg of total proteins and western blot. Band intensity was quantified by densitometry using the band detection and histogram tools of Fiji software^66^.

### Confocal microscopy

Plasmid pSB4C5-RFP was electroporated into *P. atrosepticum* SCRI 1043, and transformants were selected in LB agar with 20 mg/ml chloramphenicol. Three days post inoculation, cross sections of potato petioles were carefully taken from the inoculation site and 1 cm above or below this site. Slices of infected tubers were obtained at 0.5 and 1 cm below the inoculation site. Samples were placed on microscope slides and observed with an Olympus FV1000 confocal microscope with 10x and 20x objectives. Red fluorescent protein was excited at 488 nm using an argon laser and an HFT UV 488/543/633 nm dual dichroic excitation mirror with an NFT 490 Beam Splitter and a BP-500-530 IR emission filter for detection. The offset value was adjusted until no background fluorescence was observed. Images were obtained with LSM 510 Release Version 4.2 5P1 software (Carl Zeiss Macro- Imaging GmbH, R & D in collaboration with EMBL Heidelberg, Germany). Images were analysed with the Fiji software^66^. Infection assays and cell confocal observation of RFP-transformed bacteria were performed at least four times.

### Infection bacterial count

Sections of potato petioles or tubers at the indicated distances from the inoculation site were ground with 1 ml of 0.25x Ringer solution (38.5 mM NaCl, 1.4 mM KCl, 0.45 mM CaCl_2_, 0.59 mM NaHCO_3_, pH 7), 10^3^–10^5^ dilutions were plated onto crystal violet-pectin (CVP) plates to select for *Pectobacterium*^67^ and determine the colony-forming units (CFU). The initial bacterial suspension was also plated and the resulting CFU were used as a control. Results show the average and standard deviation of the data. Experiments with a technical duplicate were performed by triplicate.

### Swarming motility assays

The swarming phenotypes were studied as previously reported for *P. atrosepticum*^46^. Briefly, freshly prepare plates of 0.4X MacConkey agar base (Sigma) supplemented with 0.5% glycerol were inoculated with 3 μl of ~1 × 10^8^ cells. Once the drop was completely dry the plates were incubated at 28 °C in a humidity chamber for 48 h. Experiments were performed at least five times.

### Exl1 infiltration and pathogen challenge

Mature celery petioles and 4-week old leaves from *A. thaliana* strains (ecotype Columbia-0 (Col-0), or mutants *jar-1*^68^ and *eds5*^39^, and the transgenic line NahG^41^) were vacuum infiltrated with 0, 1, 3.7, 8, 10 or 20 μM of pure preparations of recombinant Exl1 (WT or mutants D83A and YWY prepared as indicated by Olarte-Lozano *et al*.^69^) and diluted in 0.002% glucose solution (mock). Experiments were incubated 24 hours at 20 °C ± 2 °C with continuous light in a humidity chamber, followed by inoculation of 6 μl of a LB suspension containing 10^8^ *P. brasiliense* BF45 cells and continuous incubation. After 24, 48 or 72 h, the incidence percentage (number of infected leaves) was calculated.

### Intracellular ROS quantification

The ROS measurement was perform as previously reported^70^. Briefly, *A. thaliana* leaves were infiltrated with the protein variants and after 5 min or 1 h were submerged in 60 mM of carboxy-2′, 7′-dichloro-dihydro-fluorescein diacetate (DCFH-DA, Sigma) incubated for 30 min at RT in the dark, followed by a distilled water rinse to remove the dye excess. Fluorescence was measured using a Leica DMR epifluorescence microscope with a GFP filter (excitation 480/40 nm, emission 527/30 nm) and quantified from the acquired images with the Fiji software^66^.

All figures were created with Power Point-Office 365 ProPlus (using the high-resolution export settings) and GIMP v 2.10.14 (https://www.gimp.org/) software.

## Supplementary information

Supplementary Information.
