# Supplementary material for: Expansin-like Exl1 from Pectobacterium is a virulence factor required for host infection, and induces a defence plant response involving ROS, and jasmonate, ethylene and salicylic acid signalling pathways in Arabidopsis thaliana
Source: Sci Rep. 2020 May 8;10:7747. doi: 10.1038/s41598-020-64529-9 (PMC7210985; doi:10.1038/s41598-020-64529-9)
Supplement: Supplementary file 1 — Supplementary Information. [file 41598_2020_64529_MOESM1_ESM.docx]

Supplementary Material

**Expansin-like Exl1 from *Pectobacterium* is a virulence factor required for host infection, and induces a defence plant response involving ROS, and jasmonate, ethylene and salicylic acid signalling pathways in *Arabidopsis thaliana***

Delia Narváez-Barragán^1^, Omar E. Tovar-Herrera^1^^§^, Martha Torres^2^, Mabel Rodriguez^1^, Sonia Humphris^3^, Ian Toth^3^, Lorenzo Segovia^1^, Mario Serrano^2^ and Claudia Martinez-Anaya^1*^

^1^Instituto de Biotecnología and ^2^Centro de Ciencias Genómicas. Universidad Nacional Autónoma de México. 62110 Cuernavaca Morelos, Mexico

^3^The James Hutton Institute, Invergowrie Dundee DD2 5DA, UK

^§^ Current Address: Department of Biomolecular Sciences, The Weizmann Institute of Science, 7610001 Rehovot, Israel.

**i. Supplementary Material and Methods**

**Biofilm quantification**

Biofilm was quantified as described in (1). Briefly, overnight cultures of *P. atrosepticum* SCRI 1043 wild type and *Δexl1* were adjusted to 1.2 OD_600 nm_ and diluted 1:100 with LB broth. In a 96-well plate, 100 μl of the dilution was added per well and incubated at 27ºC for 48 h in a humidity chamber. After this period, plates were washed twice with distillate water. Biofilm was stained with 125 μl of 0.1% crystal violet per well and discarded after 10 min; then plates were washed three times. Biofilm was solubilised with 200 μl 95% EtOH. After 15 min, 100 μl of the solution were transferred into a clean plate. Absorbance was quantified at 570 nm in a Bio-Rad plate reader.

***In vitro* enzyme activity assay**

*P. atrosepticum* wild type or *Δexl1* cells were grow overnight in LB broth, then centrifugated and resuspended in 1 M MgSO4 and adjusted to 10^4^ cells/ml. One 10-μl drop of this cell suspension was spotted in the middle of plates containing different substrates and incubated at 27ºC for 10 days. For pectinase activity plates contained 0.1% yeast extract, 0.1% (NH_4_)_2_SO_4_, 0.001M MgSO_4_, 0.5% glycerol, 0.5% poly galacturonic acid (PGA), 0.2X phosphate buffer and 1.6% agar. Pectinase activity was developed by flooding the plate with 7.5% Cu(CH_3_COO)_2_ for 2 hours. For cellulase activity plates contained 0.5% yeast extract, 0.1% (NH_4_)_2_SO_4_, 0.001% MgSO_4_, 0.2% glycerol, 1x phosphate buffer, 1% carboxy methyl cellulose (CMC) and 1.6% agar. Activity was observed by flooding the plate with 0.2% Congo red for 20 minutes, rinsing with 1 M NaCl for 15 minutes, and finally flooding the plates with 1 M HCl for 5 minutes. For protease activity plates contained 3% gelatine and 1.6% agar in LB media. To observe protease activity plates were flooded with 4M (NH_4_)_2_SO_4_ for 30 minutes. Activity halos diameter was measured and compared between strains. Experiments were performed by triplicate.

**Root attachment assays**

Roots of four-week old potato plants were washed with sterile distilled water, absorbing the excess with blotting paper. One gram of roots (wet weight) were submerged in 10 ml of 10^8^ cells suspension of *P. atrosepticum* wild type or *Δexl1* (diluted in PBS) for 1 h with shaking (500 osc/min). Roots were then washed three times with 20 ml of PBS and shaking for 5 min at 800 osc/min to remove non-attached bacteria, and the grinded using BIOREBA extraction bags and a Homex machine. The obtained supernatant was diluted in PBS and plated onto CVP plates (2), to count the cavities after incubation at 27ºC for 48 hours.

**Assay of bacteria viability in the presence of hydrogen peroxide (H_2_O_2_)**

Sensitivity to hydrogen peroxide of *P. atrosepticum* wild type or *Δexl1*, was assessed as described in (3) with some modifications. Cells were grown in LB broth until mid-exponential phase (between 0.2-0.4 OD_600 nm_). Then, H_2_0_2_ was added to a final concentration of 750 μM. Samples were taken after 0, 5, 10 and 15 min incubation in the presence of H_2_O_2_. At which time points serial dilutions were plated onto LB agar and incubated overnight at 30ºC. Cell survival was calculated by comparing CFUs before and after treatment with H_2_O_2_

**Growth in hyperosmotic medium**

Overnight cultures of *P. atrosepticum* wild type or *Δexl1* strains were adjusted to 0.5 OD_600 nm_ and 5 μl of cell suspension dilutions from 10^0^ to 10^-4^ were placed in LB agar added with 2.5% or 5% NaCl. Plates were incubated for 24 or 48 h at 30°C.

**Swimming assay**

LB plates (0.3% agar) were inoculated with a fresh colony of *P. atrosepticum* wild type or *Δexl1* using a sterile pipette tip. Plates were incubated at 27ºC for 3 days. Swimming was compared between strains by measuring the halo of swimming cells. Experiments were performed 6 times.

**ii. Supplementary Table 1.** List of oligonucleotides used in this work

| **Gene-Experiment** | **Primer name** | **Sequence (5’ 🡪 3’)** | **Reference** |
| --- | --- | --- | --- |
| *Exl1*- mutation (upstream region) | XbaI-fw | aaatctagaccagcgagacatccgcagtc | This study |
|  | HindIII-rev | aaaaagcttgtttcacatccaatatgattc | “ |
| *Exl1*- mutation (downstream region) | XhoI-fw | aaactcgagtcgcccacaacttgagggac | “ |
|  | ApaI-rev | aaagggcccgcacgctggctgatgtcggc | “ |
| *Exl1*-complementation and overexpression | XhoI-fw | ctcgagtgcgcggccgcttaaagc | “ |
|  | SalI-rev | gttgttgtcgactaactttaagaaggagatatacat | “ |
| *RecA-* RT-qPCR | recA-fw | ggtgagctggttgatctggg | (4) |
|  | recA-rev | gcattcgctttaccctgacc | “ |
| *Exl1-* RT-qPCR | PaExl1-fw | gcggtgcaatttaggaatgt | This study |
|  | PaExl1-rev | tgacattggtggtagcgtgt | “ |
| Actin (At4g26410)- RT-qPCR | At4g26410-fw | gagctgaa^gtggcttccatgac | (5) |
|  | At4g26410-rev | ggtccgacatacccatgatcc | “ |
| PDF1.2 (At5g44420)- RT-qPCR | PDF-fw | ccaagtgggacatggtcag | (6) |
|  | PDF-rev | acttgtgtgctgggaagac | “ |
| PR4 (At3g04720) - RT-qPCR | PR4-fw | gtaccaccgcggactactgt | “ |
|  | PR4-rev | tggaggaataagcactcacg | “ |
| PR1 (At2g14610) -  RT-qPCR | PR1-fw | ttcttccctcgaaagctcaa | “ |
|  | PR1-rev | aaggcccaccagagtgtatg | “ |
| COI1 (At2g39940.1)- RT-qPCR | COI-fw | ggaagttctcgagacaaggaatg | (6) |
|  | COI-rev | acaaggcggaagtcacagag | “ |
| EDS5 (At4g39030.1)- RT-qPCR | EDS5-fw | gaatctaaacagtcgggcatacc | “ |
|  | EDS5-rev | ggggctcgagaagatcagacaga | “ |
| ZAT12 (At5g59820-s) - RT-qPCR | ZAT12-fw | atcaagtcgacggtggatgt | Referencia? |
|  | ZAT12-rev | acaaagcgtcgttgttaggc |  |

**iii) Supplementary Figures**


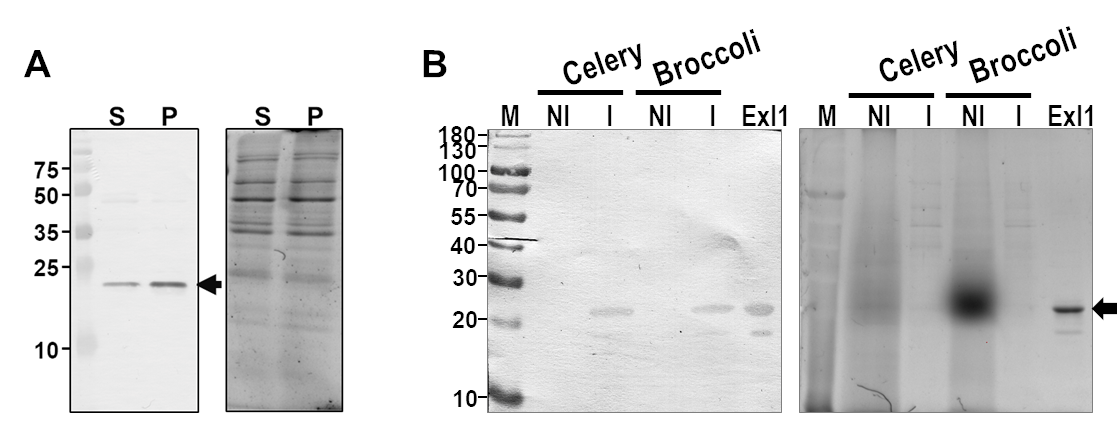


**Supplementary Figure 1.** Complete western blots (left) and protein gels visualised by 2,2,2-trichloroethanol staining (right) of Exl1 expression by *Pectobacterium* *brasiliense* infected vegetables used in Figure 1A and 1B. **A)** Western blot of protein Exl1 is observed (arrow) in the soluble (S) and insoluble (pellet, P) fractions of macerated tissue infected with *P. brasiliense* strain BF45. **B)** Plant endogenous expansins are not recognised by anti Exl1 antibody in non-infected (NI) celery or broccoli. Pure Exl1 was included as a control (arrow).


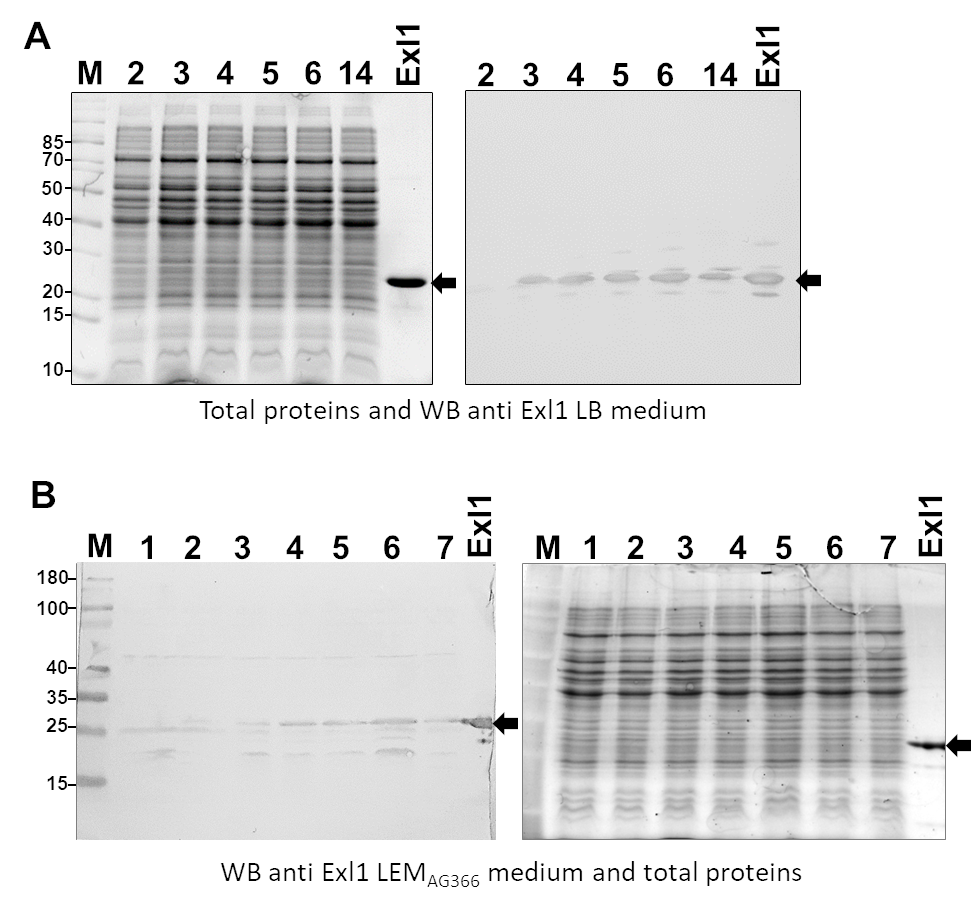


**C**


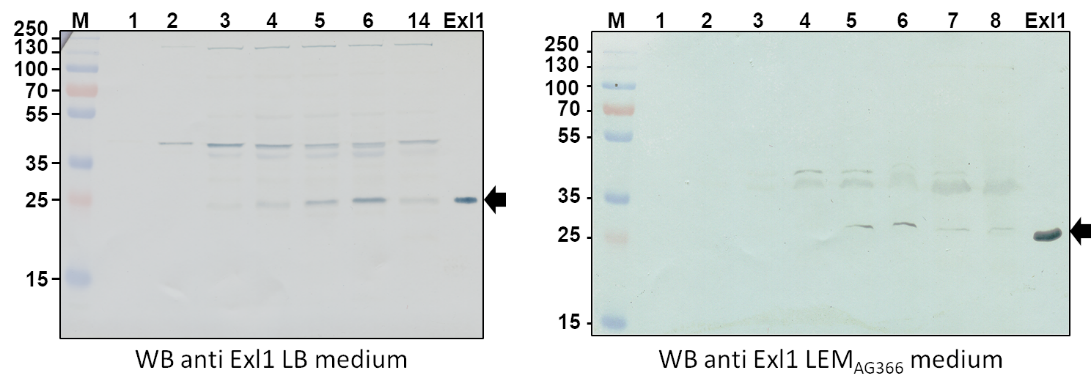


**Supplementary Figure 2.** Complete western blots and protein gels of Exl1 expression by *P.* *brasiliense* of the kinetics of expression of Exl1 in liquid LB broth (**A**, and Figure 1C in the main text); proteins were visualised by 2,2,2-trichloroethanol staining. **B)** Exl1 expression kinetics was also analysed in medium containing pectin (LEM_AG366_). **C)** Replicate experiments showing the expression of Exl1 in comparison to an unspecific band identified by the anti Exl1 antisera. Bacteria were grown in LB (right) or LEM_AG366_ (left); samples were taken at the indicated times (h). One microgram of pure Exl1 was included as a control (arrow). Bands from LEM_AG366_ cultures sometimes run above pure Exl1 obtained from *E. coli* (arrow).


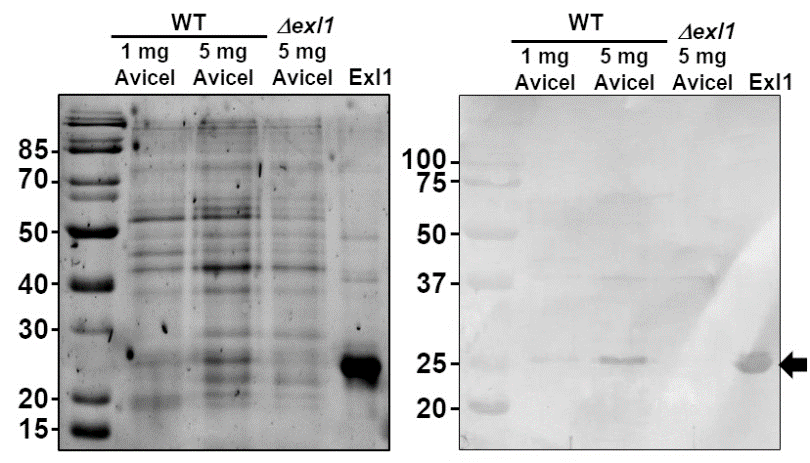


**Supplementary Figure 3.** Complete Western blot and protein gel of Exl1 pulldown from *P. atrosepticum* wild type and *Δexl1* mutant strain, shown in Figure 1D. Pulldown of Exl1 from concentrated supernatants of cultures at late exponential phase of *P. atrosepticum* wild type (WT) and *Δexl1* mutant strains. One and five micrograms of Avicel were used for pulling down as indicated. One microgram of pure Exl1 was included as a control (arrow). Protein gel was visualised by 2,2,2-trichloroethanol staining.


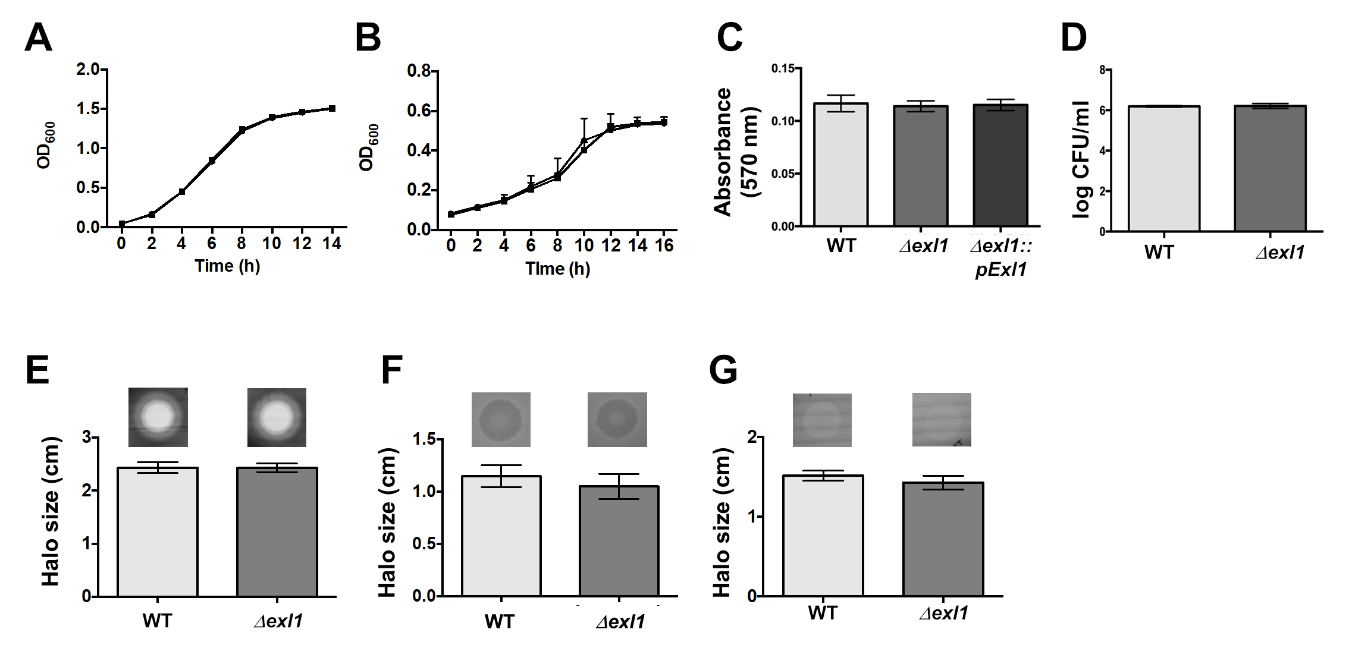


**Supplementary Figure 4. Phenotypes of *P. atrosepticum* wild type and *Δexl*1 mutant.** Growth kinetics of the strains *P. atrosepticum* wild type (circle) and *Δexl1* (square) are similar, either growing in **A)** LB broth or **B**) minimum media supplemented with pectin (LEM_AG3360_) at 30ºC at the indicated times. **C**) The *in vitro* biofilm formation of the mutant *Δexl1* (medium grey) is comparable with the *P. atrosepticum* wild type strain (light grey) and with the *Exl1* overexpressing strain (dark grey). The experiment was repeated three independent times with a total of 72 absorbance readings per strain. **D**) CFUs of bacteria attached to potato roots indicate similar capacity for root attachment for wild type and mutant strains calculated from serial dilutions of bacteria growing in CVP plates. (**E-G**) Activity of major PCWDEs in *P. atrosepticum* wild type (light grey) and *Δexl1* (dark grey) are similar. The activity was measured using plates with substrates for **E)** cellulases, **F)** proteases or **G)** pectinases. In all cases, data are the average of at least three independent experiments. Error bars correspond to the standard deviation of the experiments. No statistical differences were observed according to student’s *t* test.


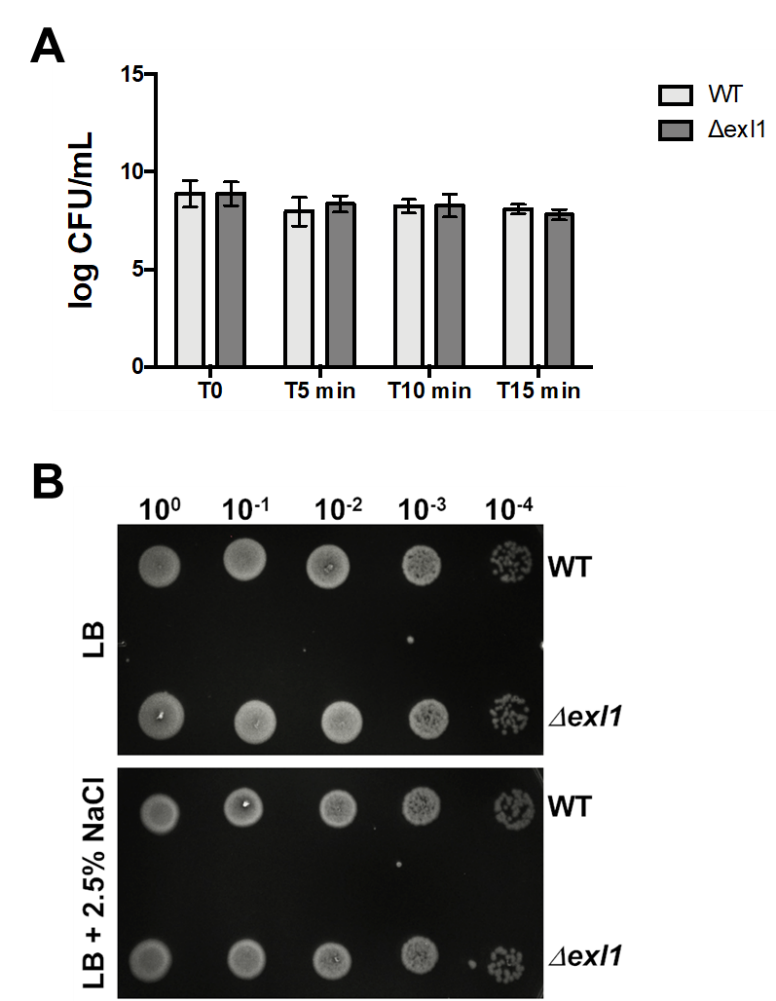


**Supplementary Figure 5. *P. atrosepticum* wild type or *Δexl1* have the same sensitivity to oxidative and hyperosmotic stress. A)** Cell count of *P. atrosepticum* wild type (light grey) and *Δexl1* (dark grey) strains that survived in the presence of H_2_O_2_ is similar. CFUs were counted at 0, 5, 10 and 15 min after H_2_0_2_ addition. Data is the average of at least three independent experiments. Error bars correspond to the standard deviation of the experiments. **B)** Cell dilutions of *P. atrosepticum* wild type and *Δexl1*grown in LB medium at 30°C for 24 h, or in LB containing 2.5% NaCl at 30°C, which under this condition both WT and *Δexl1* strains required 48 h to grow.


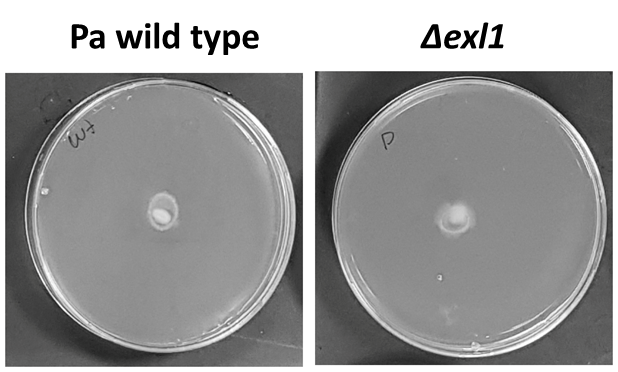

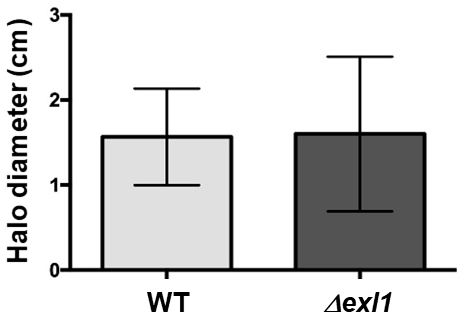


**Supplementary Figure 6. Swimming capacity of *P. atrosepticum* wild type and *Δexl1* strains.** Cells of *P. atrosepticum* wild type and *Δexl1* showed the same swimming phenotype in LB medium (0.3% agar), as the area of radial migration is comparable between both strains (n=6 plates per strain).

**References**

1. Merritt JH, Kadouri DE, O’Toole GA. Growing and analyzing static biofilms. *Curr Protoc Microbiol*. 2011;Chapter 1:Unit 1B.1. doi: 10.1002/9780471729259.mc01b01s00.

2. Hélias V, Hamon P, Huchet E, Wolf J V.D., Andrivon D. Two new effective semiselective crystal violet pectate media for isolation of *Pectobacterium* and *Dickeya*. Plant Pathol. 2012;

3. Macvanin M, Hughes D. Assays of sensitivity of antibiotic-resistant bacteria to hydrogen peroxide and measurement of catalase activity. Methods Mol Biol. 2010;

4. Takle GW, Toth IK, Brurberg MB. Evaluation of reference genes for real-time RT-PCR expression studies in the plant pathogen *Pectobacterium atrosepticum*. BMC Plant Biol. 2007;

5. Czechowski T. Genome-Wide Identification and Testing of Superior Reference Genes for Transcript Normalization in *Arabidopsis*. PLANT Physiol. 2005;

6. Hael-Conrad V, Abou-Mansour E, Díaz-Ricci JC, Métraux JP, Serrano M. The novel elicitor AsES triggers a defense response against *Botrytis cinerea* in *Arabidopsis thaliana*. Plant Sci [Internet]. 2015;241:120–7. Available from: http://dx.doi.org/10.1016/j.plantsci.2015.09.025
